# Surgical strategies in the treatment of chronic pancreatitis: An updated systematic review and meta-analysis of randomized controlled trials

**DOI:** 10.1097/MD.0000000000006220

**Published:** 2017-03-03

**Authors:** Xin Zhao, Naiqiang Cui, Ximo Wang, Yunfeng Cui

**Affiliations:** a Department of Surgery, Tianjin Nankai Hospital, Tianjin, China; b Nankai Clinical College, Tianjin Medical University, Tianjin, China.

**Keywords:** chronic pancreatitis, randomized controlled trials, surgical strategy, updated meta-analysis

## Abstract

**Background:**

Chronic pancreatitis (CP) is a common and frequently occurring disease. Pancreaticoduodenectomy (PD), pylorus-preserving pancreaticoduodenectomy (PPPD), and duodenum-preserving pancreatic head resection (DPPHR) are important treatment options for patients with chronic pancreatitis. The Beger and Frey procedures are 2 main duodenum-preserving techniques in duodenum-preserving pancreatic head resection (DPPHR) strategies. We conducted this systematic review and meta-analysis to compare the clinical efficacy of DPPHR versus PD, the Beger procedure versus PD, the Frey procedure versus PD, and the Beger procedure versus the Frey procedure in the treatment of pancreatitis. The optimal surgical option for chronic pancreatitis is still under debate. The aim of this systematic review and meta-analysis was to evaluate the clinical efficacy of different surgical strategies for chronic pancreatitis.

**Methods:**

Five databases (PubMed, Medline, SinoMed, Embase, and Cochrane Library) were searched with the limitations of human subjects and randomized controlled trials (RCTs) text. Data were extracted by 2 of the coauthors independently and analyzed using the RevMan statistical software, version 5.3. Weighted mean differences (WMDs), risk ratios (RRs), and 95% confidence intervals (CIs) were calculated. Cochrane Collaboration's Risk of Bias Tool was used to assess the risk of bias.

**Results:**

Seven studies involving a total of 385 patients who underwent the surgical treatments were assessed. The methodological quality of the trials ranged from low to moderate and included PD (n = 134) and DPPHR (n = 251 [Beger procedure = 100; Frey procedure = 109; Beger or Frey procedure = 42]). There were no significant differences between DPPHR and PD in post-operation mortality (RR = 2.89, 95% CI = 0.31–26.87, *P* = 0.36), pain relief (RR = 1.09, 95% CI = 0.94–1.25, *P* = 0.26), exocrine insufficiency (follow-up time > 60 months: RR = 0.91, 95% CI = 0.72–1.15, *P* = 0.41), and endocrine insufficiency (RR = 0.75, 95% CI = 0.52–1.08, *P* = 0.12). Concerning the follow-up time < 60 months, the DPPHR group had better results of exocrine insufficiency (RR = 0.22, 95% CI = 0.08–0.62, *P* = 0.04). However, operation time (*P* < 0.0001), blood transfusion (*P* = 0.02), hospital stay (*P* = 0.0002), postoperation morbidity (*P* = 0.0007), weight gain (*P* < 0.00001), quality of life (P = 0.01), and occupational rehabilitation (*P* = 0.007) were significantly better for patients who underwent the DPPHR procedure compared with the PD procedure. The comparison results of the Frey procedure and PD showed that both procedures had an equal effect in the pain relief, postoperation mortality, exocrine and endocrine function, and quality of life (QoL) (*P* > 0.05), whereas patients who underwent the Frey procedure had significantly reduced operative times (*P* < 0.05) and less blood transfusions (*P* < 0.05). Comparing the Beger procedure to the PD procedure, there were no significant differences in hospital stay, blood transfusion, postoperation morbidity or mortality, pain relief, weight gain, exocrine insufficiency, and occupational rehabilitation (*P* > 0.05). Two studies comparing the Beger and Frey procedures showed no differences in postoperative morbidity, pain relief, exocrine insufficiency, and quality of life (*P* > 0.05). In terms of operative time, blood transfusion, hospital stay, postoperation morbidity, weight gain, quality of life, and occupational rehabilitation, the results also favored duodenum-preserving pancreatic head resection (DPPHR) strategies.

**Conclusion:**

All procedures are equally effective for the management of pain, postoperation morbidity, exocrine insufficiency, and endocrine insufficiency for chronic pancreatitis. Improved short- and long-term outcomes, including operative time, blood transfusion, hospital stay, quality of life, weight gain, and occupational rehabilitation make DPPHR a more favorable surgical strategy for patients with chronic pancreatitis. Further, relevant trails are eager to prove these findings.

## 1 Introduction

Chronic pancreatitis is a type of chronic and persistently occurring pancreatic inflammatory damage that may cause fibrosis, dilatation of pancreatic duct, pancreatic duct stones or calcification and irreversible morphological changes of pancreas, as well as intractable pain and permanent losses of exocrine and endocrine function.^[1,2]^ Chronic pancreatitis carries a high burden of morbidity because of its long duration and recurrent attacks. Currently, the main treatment methods for chronic pancreatitis are focused on pain management, the management of complications (i.e., pseudocysts), and the correction of pancreatic insufficiency.^[3]^ Intractable abdominal pain is the main surgical indication for chronic pancreatitis. Pancreatic hyperplasia is also a potential carcinogen. Therefore, surgical treatment can improve the quality of life of patients not only by relieving pain and retaining the internal and external secretion of pancreatic function but also by effectively removing the risk factors for cancer.^[4]^ One recent meta-analysis compared endoscopic and surgical interventions in chronic pancreatitis and indicated that surgery is a promising approach in the treatment of chronic pancreatitis and had an obvious advantage for pain relief, which is difficult to achieve with medical treatment.^[5]^ Many factors such as disease location (head, body, tail of the pancreas, or diffuse disease) or the suspicion of cancer can often affect the surgical approach. At present, the main operative modes for the treatment of chronic pancreatitis are pancreaticoduodenectomy (PD) and duodenum-preserving pancreatic head resection (DPPHR).^[6]^ PD includes the Whipple procedure and the pylorus-preserving pancreaticoduodenectomy (PPPD). DPPHR includes both the Beger and Frey procedures.^[7–10]^ Traditionally, pancreatoduodenectomy with or without preservation of the pylorus is the main operative approach for the treatment of chronic pancreatitis.^[11]^ However, this complex procedure seems excessive for benign conditions because high morbidity and endocrine insufficiency limit its clinical application.^[12]^ Surgical approaches have undergone a transformation over the past few years, with the pancreatic head resection that has become universally appreciated as the nidus of chronic inflammation.^[13]^

Currently, the available evidence regarding the clinical significance of PD and DPPHR for the treatment of chronic pancreatitis is limited due to the small number of patients in reported studies.^[14]^ However, it is important to determine the optimal surgical strategy for patients with CP and there is currently no consensus on the method of pancreatic head resection in people with chronic pancreatitis. In 2010, a review of the surgical treatment of chronic pancreatitis concluded that duodenum-preserving pancreatic head resection (DPPHR) offered outcomes as effective as pancreaticoduodenectomy (PD); however, DPPHR appeared to provide better outcomes in terms of morbidity and mortality.^[15]^ A recent 2016 meta-analysis comparing DPPHR and PD yielded similar results, which reached the same conclusion and suggested that DPPHR may result in a shorter hospital stay, but there was no evidence of any differences in mortality, adverse events, or quality of life between the 2 procedures.^[16]^

Pancreatoduodenectomy has long been regarded as the standard surgical approach for the treatment of chronic pancreatitis. However, this complex procedure also has some disadvantages. Since none of stomach, duodenum or the common bile duct are directly involved in the inflammatory processes of the pancreas, the PD procedure might lead to over-treatment. To further assess the advantages and disadvantages of these surgical procedures and provide guidance for clinical decision making, we conducted a more thorough literature search and updated meta-analysis to compare the different surgical strategies in the treatment of chronic pancreatitis.

## 2 Methods

Ethical approval or patient consent was not required since the present study was a review of previous published literatures.

### 2.1 Search strategy and study selection criteria

A computerized search was conducted from inception to June 2016 with the PubMed, Medline, SinoMed, Embase, and Cochrane Library databases. The databases were queried for eligible literature using combinations of the following keywords: “chronic pancreatitis,” “pancreatitis,” “pancreaticoduodenectomy,” “pylorus-preserving,” “duodenum-preserving,” “pylorus-preserving pancreaticoduodenectomy,” “Beger procedure,” “Frey procedure,” “duodenum-preserving pancreaticoduodenectomy,” “Whipple procedure,” and “pancreatic head resection.” The search was limited to human subjects. There was no language limitation. The titles and abstracts of potentially relevant studies were reviewed. Additionally, bibliographies of all included articles were screened for potentially relevant studies. Full-text articles were obtained for detailed evaluation and eligible studies were included in the systematic review.

The inclusion criteria were as follows: (1) the study populations were patients diagnosed with CP who were randomly allocated to undergo either a DPPHR or a PD procedure; (2) the aims of the trial were to compare the effectiveness of DPPHR (described by Beger, Frey, or Bern et al) with either a PD or Whipple procedure or the Beger versus the Frey procedure; (3) the trial was a randomized controlled trial; and (4) the postoperative follow-up time was not less than 12 months. If a study generated multiple publications, but the median follow-up time was different, then the relevant parameters of the follow-up interval were compared. The comprehensive relevant parameters were integrated into 1 article.

### 2.2 Data extraction and quality assessment

Extracted data included the characteristics of the eligible studies: author, country, study design, sample size, sex, mean age, history of symptoms, mean follow-up time, evaluation of pain, and quality of life (QoL). Two reviewers independently extracted and checked the research data to ensure consistency. The quality of trials that were designed with control and treatment groups were assessed using Review Manager (Version 5.3; The Cochrane Collaboration, Oxford, UK). The risk of bias for RCTs was evaluated with the Cochrane Collaboration's Risk of Bias Tool. Seven parameters were used to evaluate the quality of each included study: random sequence generation, allocation concealment, blinding, incomplete outcome data, selective outcome reporting, and other risks. Items were judged as “low risk,” “unclear risk,” or “high risk.”

### 2.3 Outcome measures

The short-term outcomes included operative time, blood transfusion, length of hospital stay, postoperative mortality, and postoperative morbidity. Long-term outcomes included mortality, exocrine insufficiency, endocrine insufficiency, pain relief, quality of life, and occupational rehabilitation. The quantification of pain relief had to be based on the pain score or other similar criteria. Moreover, long-term quality of life was assessed using the functional and symptom scale scores of surviving patients. Both scales included global health, physical functioning, emotional functioning, working ability, social functioning, cognitive functioning, pain, fatigue, nausea, loss of appetite, diarrhea, financial strain, fever, itching, treatment strain, and so on, that were analyzed with the available data.

### 2.4 Statistical methods

Using a systematic review, a meta-analysis was conducted using the software Review Manager 5.3 (Cochrane Collaboration, http://tech.cochrane.org/revman/download). For dichotomous outcomes, risk ratios (RRs) and 95% confidence intervals (CI) were calculated using extracted data, whereas weighted mean differences (WMDs) and 95% CI were used for continuous outcomes. Heterogeneity was assessed using the *Q* test and *I*^2^. Statistical significance was set at *P* < 0.05. If there was significant heterogeneity (*P* ≦ 0.05, *I*^2^ ≧ 50%), a random-effects model was adopted. Otherwise, fixed-effects models were applied if there was no significant heterogeneity (*P* ≧ 0.05, *I*^2^ ≦ 50%). When the interquartile range (IQR) and medians were provided instead of standard deviations (SD), we converted the data to estimate standard deviation using Hozo's algorithm.^[17]^

## 3 Results

### 3.1 Data extraction

**Figure 1:**
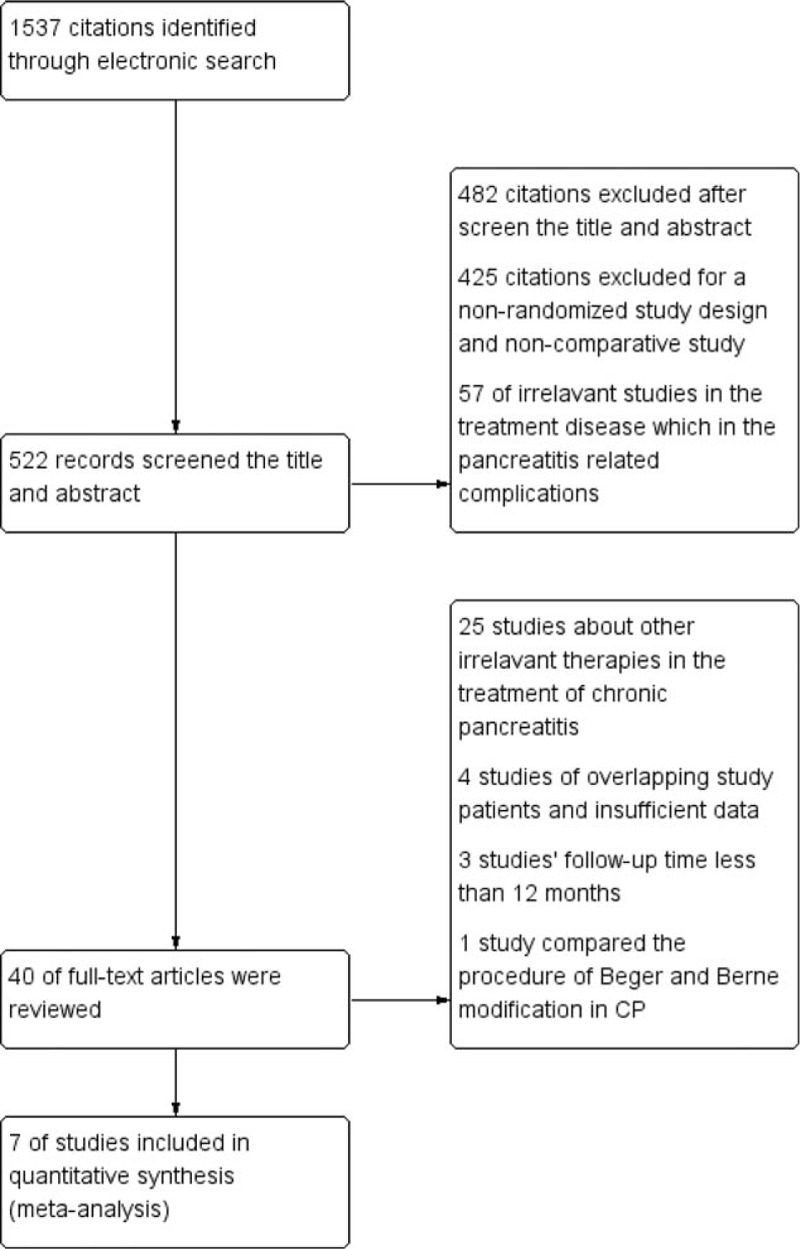
Flow diagram for the selection of randomized controlled trials included in the meta-analysis.

A total of 1537 articles were collected and after removing 1058 duplicative studies and 482 papers that were not relevant to the subject and that were excluded based on review of the titles and abstracts, 40 full-text articles were assessed for eligibility. Ultimately, 7 clinical studies satisfied the inclusion requirements.^[18–24]^ A detailed study flow diagram is shown in Fig. 1.

### 3.2 Description of studies

In accordance with the search strategy and study selection criteria, 7 trials were identified for inclusion in this meta-analysis. The characteristics of the included studies are presented in Table 1. The 7 assessed RCTs studies included 6 in English^[18,20–24]^ and 1 in German.^[19]^ The included studies were conducted from 1995 to 2016 and described 385 patients with chronic pancreatitis who underwent surgery. Overall, 134 patients underwent the PD procedure and 251 patients underwent the DPPHR (Beger or Frey procedure). The methodological quality of the trials ranged from low to moderate and most (65.71%, 253/385) of the patients were male. The median or mean age ranged from 41 to 48 years. The average follow-up duration ranged from 12 to 168 months. Short-term outcomes included operative time, blood transfusion, mortality, morbidity, and length of hospital stay. Long-term outcomes included abdominal pain relief, pancreatic endocrine (the presence of diabetes) and exocrine insufficiencies (the presence of steatorrhea, the need for oral pancreatic enzyme supplementation or assessment using a functional test), and quality of life (QoL), weight gain, and occupational rehabilitation. Pain relief was evaluated by a published pain score or other similar indices.^[25]^ Quality of life was evaluated by the European Organization for Research and Treatment of Cancer's Quality of Life Questionnaire (EORTC-QLQ).^[26]^ Both the functional and symptom scale scores were assessed in this study and were available in the data.

**Table 1:**
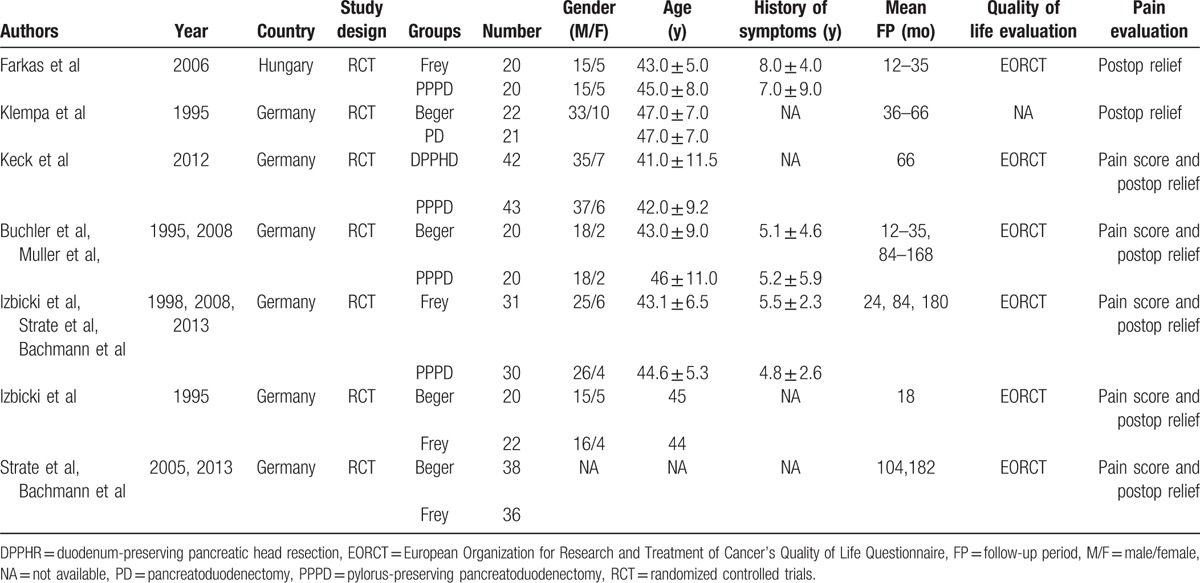
Characteristics of studies included and clinical outcomes of the study population.

Among the eligible studies, 5 studies compared DPPHR (Beger or Frey) with PD^[18–22]^ and 2 studies compared the Beger with Frey procedures.^[23,24]^ Among the studies, Strate et al,^[27]^ Izbicki et al,^[22]^ and Bachmann et al^[28]^ were from the same center of the same study, but the median follow-up was 2 years to 15 years. Muller et al^[29]^ and Buchler et al^[21]^ were also from the same center of the same study, but the median follow-up was 1 year and 7 to 14 years, respectively. In this meta-analysis, the related parameters of the follow-up interval were compared. The data from 2 or 3 studies were considered as a document included in this analysis.

### 3.3 Methodological assessment of study quality

**Figure 2:**
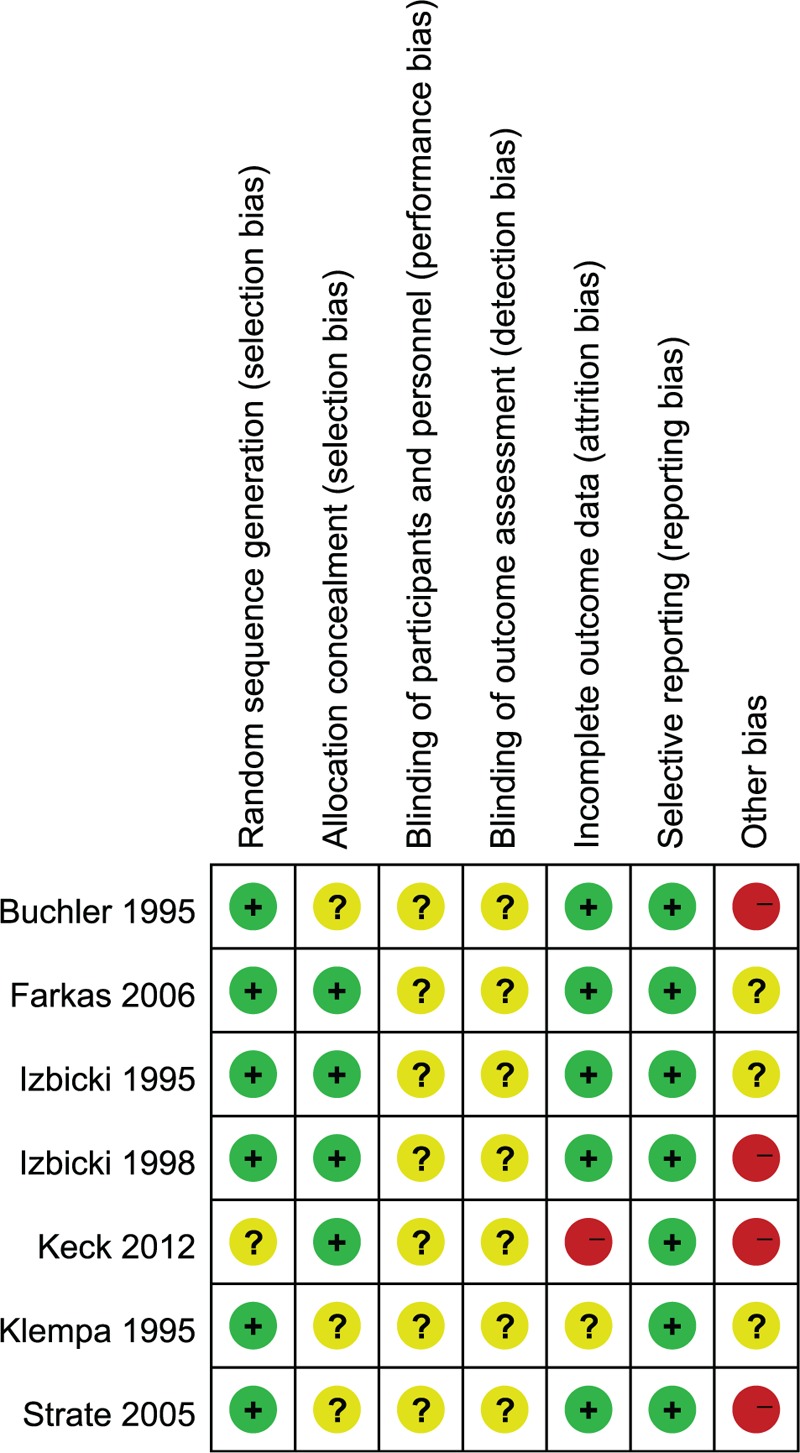
Risk of bias summary: this risk of bias tool incorporated the assessment of randomization (sequence generation and allocation concealment), blinding (participants and outcome assessors), incomplete outcome data, selective outcome reporting, and other risks of bias. The items were judged as “low risk,” “unclear risk,” or “high risk.” Red means “high risk,” green means “low risk,” and yellow means “unclear risk.”

The methodological quality assessment of the 7 included studies is presented in Fig. 2. The quality of these studies was low to moderate. All enrolled trials were RCTs. Only Izbicki et al^[22]^ described that randomization was performed using a list of random digits that were made available during surgery as coded cards that were sealed in envelopes. None of the 6 remaining studies reported the details of the random allocation of patients. Two trials provided further information to describe allocation concealment and allocation sequence generation and were at a low risk of selection bias.^[22,23]^ None of the included studies mentioned blinded status as it was impossible to the blind surgeons who performed the procedures. All 7 trials reported all important outcomes and had a low risk of reporting bias. As 2 participants were lost to follow-up, there was a high risk of attrition bias in the Keck et al^[20]^ study. The sample size and procedures with different follow-up times gave rise to high risks of selection and measurement bias, which may have affected the results.

### 3.4 Meta-analysis results

#### 3.4.1 Short-term outcomes: operative time, blood transfusion, and length of hospital stay

##### 3.4.1.1 DPPHR vs PD

**Figure 3:**
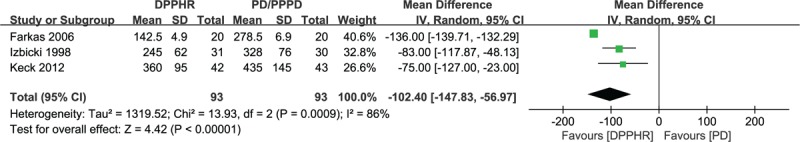
Forest plot of randomized controlled trials of duodenum-preserving pancreatic head resection versus pancreaticoduodenectomy in operative time.

**Figure 4:**
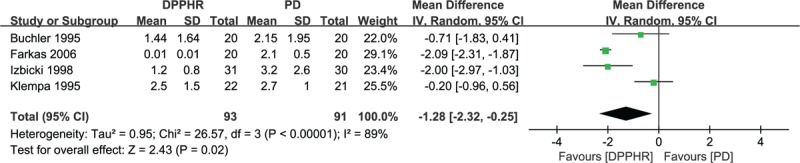
Forest plot of randomized controlled trials of duodenum-preserving pancreatic head resection versus pancreaticoduodenectomy in blood transfusion.

**Figure 5:**
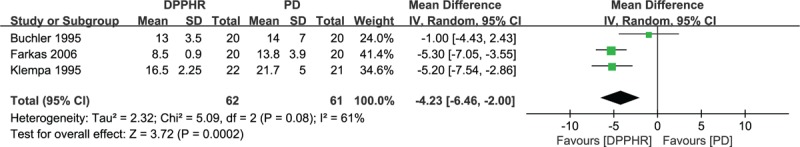
Forest plot of randomized controlled trials of duodenum-preserving pancreatic head resection versus pancreaticoduodenectomy in length of hospital stay.

Five randomized controlled studies included data on perioperative clinical outcome parameters.^[18–22]^ These studies compared the DPPHR procedure (n = 93) with pancreatoduodenectomy (n = 93) with median or mean operative times that ranged from 142.5 to 435 minutes,^[18,20,22]^ median or mean blood transfusions that ranged from 1.2 to 3.2 units,^[19,21,22]^ and median or mean hospital stays that ranged from 8.5 to 21.7 days.^[18,19,21]^ High heterogeneity was found, so we used a random-effects model to pool the data. There were obvious differences between the 2 groups in operative time (WMD = –102.40, 95%CI = –147.83 to –56.97, *P* < 0.00001), blood transfusion (WMD = –1.28, 95% CI = –2.23 to –0.25, *P* = 0.02), and length of hospital stay (WMD = –4.23, 95% CI = –6.46 to –2.00, *P* = 0.0002). The DPPHR group had a shorter operative time, less blood transfusions, and a shorter length of hospital stay compared with the pancreatoduodenectomy group (Figs. 3–5).

##### 3.4.1.2 The Frey procedure vs PD

**Figure 6:**
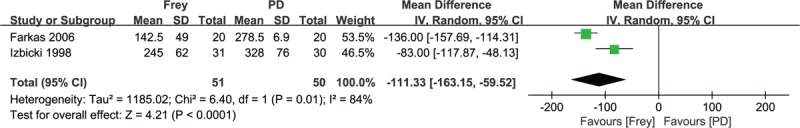
Forest plot of randomized controlled trials of the Frey procedure versus pancreaticoduodenectomy in operative time.

**Figure 7:**
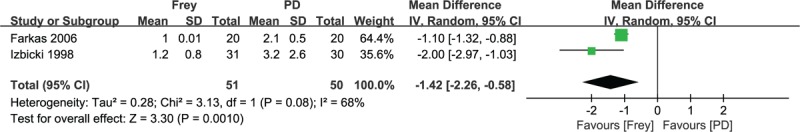
Forest plot of randomized controlled trials of the Frey procedure versus pancreaticoduodenectomy in blood transfusion.

Among the included studies, 2 randomized controlled studies (RCTs) compared the Frey procedure (n = 51) with pancreatoduodenectomy (n = 50) in operative time and blood transfusions. The Frey group had a shorter operative time (WMD = −111.33, 95%CI = −163.15 to −59.52, *P* < 0.0001) and less units of blood transfused (WMD = −1.42, 95%CI = −2.26 to −0.58, *P* = 0.001) (Figs. 6 and 7).^[18,22]^

##### 3.4.1.3 The Beger procedure vs PD

**Figure 8:**
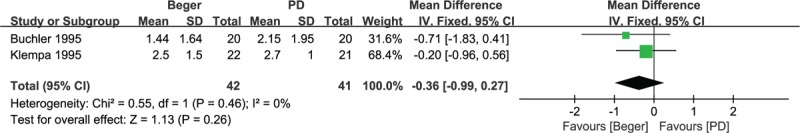
Forest plot of randomized controlled trials of the Beger procedure versus pancreaticoduodenectomy in blood transfusion.

**Figure 9:**
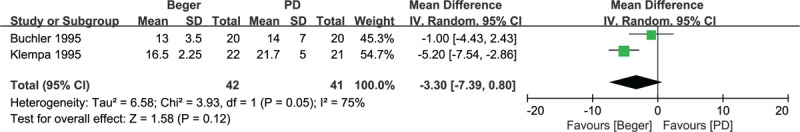
Forest plot of randomized controlled trials of the Beger procedure versus pancreaticoduodenectomy in length of hospital stay.

Two studies (RCTs) compared the Beger procedure (n = 42) with pancreatoduodenectomy (n = 41) in blood transfusion and hospital stay.^[19,21]^ Low heterogeneity among the studies was found, so we used the fixed-effects model to pool the data on blood transfusion. When compared to hospital stay, the *I*^2^ = 75%, which required a random-effects model that was adopted for analysis. There were no obvious differences between the Beger procedure and PD in blood transfusion (WMD = 0.36, 95% CI = −0.99 to 0.27, *P* = 0.26) and hospital stay (WMD = −3.3, 95% CI = −7.39 to 0.8, *P* = 0.12). Given that the horizontal block lies to the left of the vertical line, it indicates that the Beger procedure may have less units of blood transfused and shorter hospital stay than PD (Figs. 8 and 9).

#### 3.4.2 Short-term outcomes: postoperative mortality

**Figure 10:**
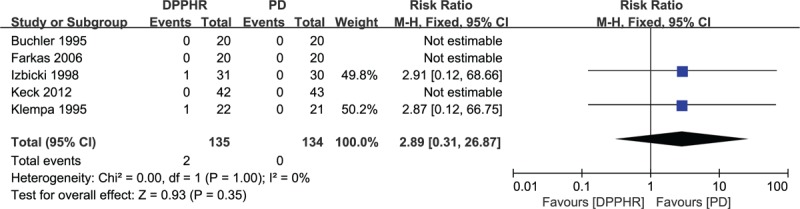
Forest plot of randomized controlled trials of duodenum-preserving pancreatic head resection versus pancreaticoduodenectomy in postoperative mortality.

Five studies reported the postoperative mortality after either a DPPHR or a PD procedure.^[18–22]^ Two out of 135 (1.48%) patients in the DPPHR group and zero patients in the PD group died during the follow-up period. One study described that a single patient in the Frey group died of cardiopulmonary failure secondary to a myocardial infarction. Low heterogeneity among the studies was revealed (*I*^2^ = 0%), so a fixed-effects model was adopted. A pooled analysis revealed that there were no significant differences between the DPPHR and PD groups in postoperative mortality (RR = 2.89, 95% CI = 0.31–26.87, *P* = 0.35) (Fig. 10).

#### 3.4.3 Short-term outcomes: postoperative morbidity

**Figure 11:**
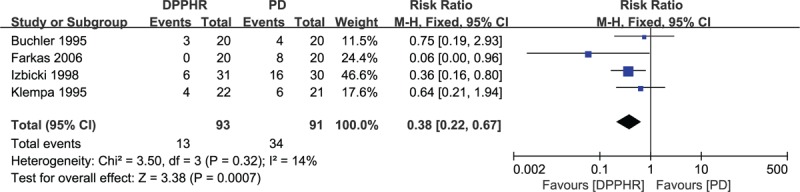
Forest plot of randomized controlled trials of duodenum-preserving pancreatic head resection versus pancreaticoduodenectomy in postoperative morbidity.

Four studies reported morbidity after either a DPPHR or a PD procedure.^[18,19,21,22]^ Twenty-three out of 93 (24.73%) patients in the DPPHR group and 40 out of 91 (43.95%) patients in the PD group had a morbidity during the follow-up period. There was low heterogeneity among the studies (*I*^2^ = 14%), so a fixed-effects model was adopted. There were significant differences between DPPHR and PD in postoperative morbidity (RR = 0.38, 95% CI = 0.22–0.67, *P* = 0.0007) (Fig. 11).

#### 3.4.4 Long-term outcomes: mortality

**Figure 12:**
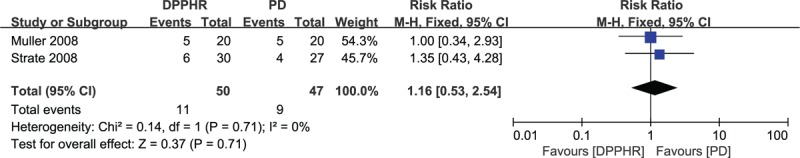
Forest plot of randomized controlled trials of duodenum-preserving pancreatic head resection versus pancreaticoduodenectomy in mortality.

Approximately 70% to 95% of patients were alive at 5 years in the various trials; however, only 2 studies had available data. Eleven out of 50 (22%) patients in the DPPHR group and 9 out of 47 (19%) patients in the PD group died during the 7 year follow-up period. Long-term mortality was not significantly different in the DPPHR group so a fixed-effect model (RR = 1.16, 95% CI = 0.53–2.54, *P* = 0.71) was used (Fig. 12). In total, 20 patients died of causes unrelated to chronic pancreatitis, and the main reasons for death included decompensated cirrhosis (3), myocardial infarction (3), renal insufficiency (1), mesenteric infarction (1), sepsis related to aspiration pneumonia (2), lung cancer (1), plasmacytoma (1), oropharyngeal carcinoma (1), and 6 of the patients who died had continuous alcohol abuse. In 7 patients, the reasons for death were unclear.

#### 3.4.5 Long-term outcomes: exocrine and endocrine insufficiency

##### 3.4.5.1 DPPHR vs PD

Data regarding exocrine insufficiency was available in 5 trials.^[18,19,22,27,29]^ There were no significant differences found in the induction of exocrine insufficiency between the DPPHR and PD groups (*I*^2^ = 39.6%, RR = 0.56, 95% CI = 0.29–1.08, *P* = 0.20). A subgroup analysis was made to describe the results of exocrine and endocrine insufficiencies, as 2 studies had follow-up times > 60 months,^[27,29]^ whereas 3 studies had follow-up times < 60 months.^[18,19,22]^ With follow-up times that were < 60 months, significant differences were found in exocrine insufficiency between the 2 groups (*I*^2^ = 52%, RR = 0.22, 95% CI = 0.08–10.62, *P* = 0.04). In the analysis of follow-up times that were > 60 months, there were no obvious differences in exocrine insufficiency between the 2 groups (*I*^2^ = 0%, RR = 0.91, 95% CI = 0.72–1.15, *P* = 0.41).

Five included studies^[18,20,22,27,29]^ provided data on endocrine insufficiency, which included 110 cases in the DPPHR group and 110 cases in the PD group. Forty-one out of 110 (37.27%) patients who underwent the DPPHR procedure appeared to have symptoms of endocrine insufficiency, whereas 51 out of 110 (46.36%) patients who underwent the PD procedure appeared to have symptoms of endocrine insufficiency. A pooled analysis with durations of follow-up < 60 months or > 60 months showed that there were no significant differences between the 2 groups in the induction of endocrine insufficiency (RR = 0.86, 95% CI = 0.55–1.34, *P* = 0.51; RR = 0.75, 95% CI = 0.52–1.08, *P* = 0.12). Taking into account that the horizontal block lies to the right of the vertical line, the upper confidence limit for the RR barely exceeds 1.0, the *P*-value is close to 0.05 for this primary efficacy outcome, these results indicate that the potential efficacy in the avoidance of endocrine insufficiency means that the DPPHR procedure may be a better choice. Results are showed in Table 2.

**Table 2:**
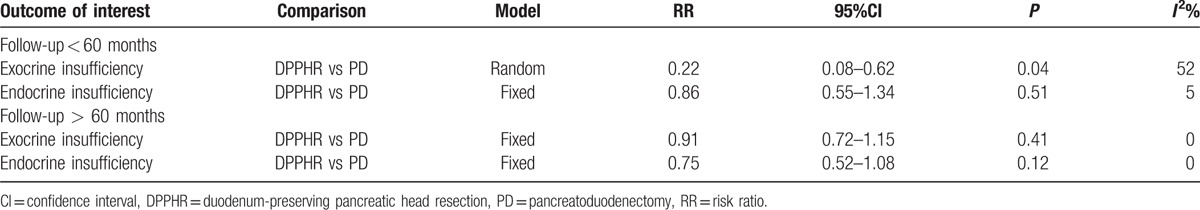
Results of exocrine and endocrine insufficiency of surviving patients.

#### 3.4.6 Long-term outcomes: pain relief

All 7 included studies reported data on pain relief. A subgroup analysis was conducted on different types of DPPHR procedures (Frey vs PD and Beger vs PD).^[23,24]^ The pain score parameters included the pain visual analog scale, the frequency of pain, analgesic medications, the pain score, and the inability to work that were compared between the 2 procedures. A pooled analysis revealed that there were no differences that were detected between the 2 groups. The outcomes of pain score parameters are shown in Table 3.

**Table 3:**
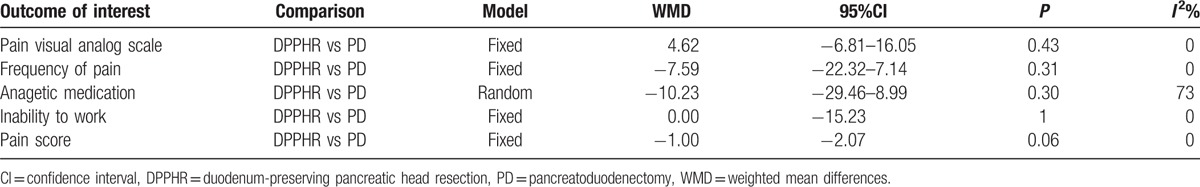
Results of long-term follow-up (14 years or 15 years) pain scores of surviving patients.

##### 3.4.6.1 DPPHR vs PD

Pain relief was reported in 99 out of 129 (76.74%) and 91 out of 129 (70.54%) respectively. Low heterogeneity was revealed among the studies (*I*^2^ = 14%), so a fixed-effects model was used. A pooled analysis revealed that there were no significant differences in pain relief between the DPPHR and PD groups (RR = 1.09, 95% CI = 0.94–1.25, *P* = 0.26) (Table 4).

**Table 4:**
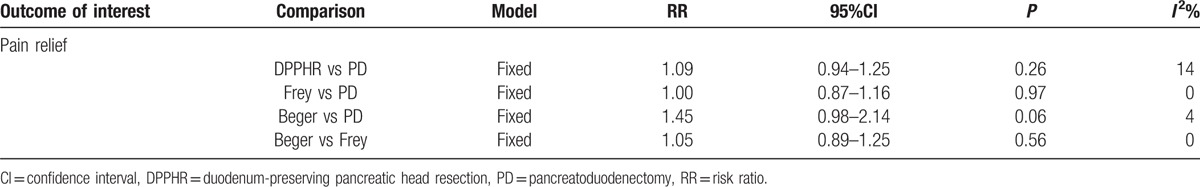
Results of pain relief of surviving patients.

##### 3.4.6.2 Frey/Beger vs PD

A subgroup analysis compared the Frey and Beger procedures with PD, which produced the same results in that there were no obvious differences between the Frey or Beger procedures compared to PD in terms of pain relief (RR = 1.00, 95% CI = 0.87–1.16, *P* = 0.97, RR = 1.45, 95% CI = 0.98–2.14, *P* = 0.06). (Table 4)

##### 3.4.6.3 Beger vs Frey procedures

Two included studies compared the Beger with the Frey procedure in terms of pain relief. A pooled analysis showed that there were no obvious differences between the Beger and the Frey procedures (RR = 1.05, 95% CI = 0.89–1.25, *P* = 0.56, *I*^2^ = 0%) (Table 4).

#### 3.4.7 Long-term outcomes: quality of life

##### 3.4.7.1 DPPHR vs PD

**Figure 13:**
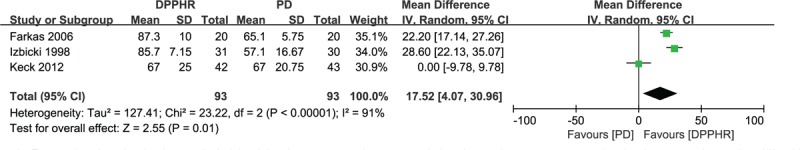
Forest plot of randomized controlled trials of duodenum-preserving pancreatic head resection versus pancreaticoduodenectomy for quality of life with the follow-up time < 60 months.

**Figure 14:**
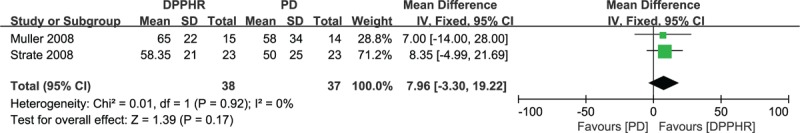
Forest plot of randomized controlled trials of duodenum-preserving pancreatic head resection versus pancreaticoduodenectomy for quality of life with the follow-up time > 60 months.

Of the 3 trials that reported data on quality of life, each used the European Organization for Research and Treatment of Cancer (EORTC) Quality-of-Life Questionnaire (QLQ) to assess the quality of life. Among 6 studies, there were 2 trials from the same research team and population but the follow-up period was different; therefore, the data were analyzed.^[18,20,22]^ A subgroup analysis was conducted with different follow-up times between the 2 groups. The analysis of DPPHR and PD procedures indicated that the patients in the DPPHR group had a significant and higher quality of life (WMD = 17.52, 95% CI = 4.07–30.96, *P* = 0.01). With the data provided for the analysis of quality of life that had a follow-up time of > 60 months, a pooled analysis showed that there were no significant differences between the 2 groups (WMD = 7.96, 95% CI = –3.30 to 19.22, *P* = 0.17).^[27,29]^ This different conclusion indicates that the period of follow-up may affect the results, which will be discussed later in the manuscript. Overall, the analysis of the results from 5 studies on quality of life showed that the DPPHR procedure significantly improved the quality of life compared to the PD group (Figs. 13 and 14). In a more detailed analysis of functional and symptom scale scores in surviving patients, the DPPHR offered additional advantages over the PD/PPPD procedures, and included a significantly reduced incidence of diarrhea and fatigue. Overall, analyzing the patients with follow-up periods of more than 10 years revealed obviously better results after the DPPHR procedure in terms of working ability, physical status, loss of appetite, loss of body weight, and financial strain. The results of different follow-up periods (less than or equal to 5 years, between 5 and 10 years, or more than 10 years) of functional and symptom scale scores in surviving patients are shown in Table 5.

**Table 5:**
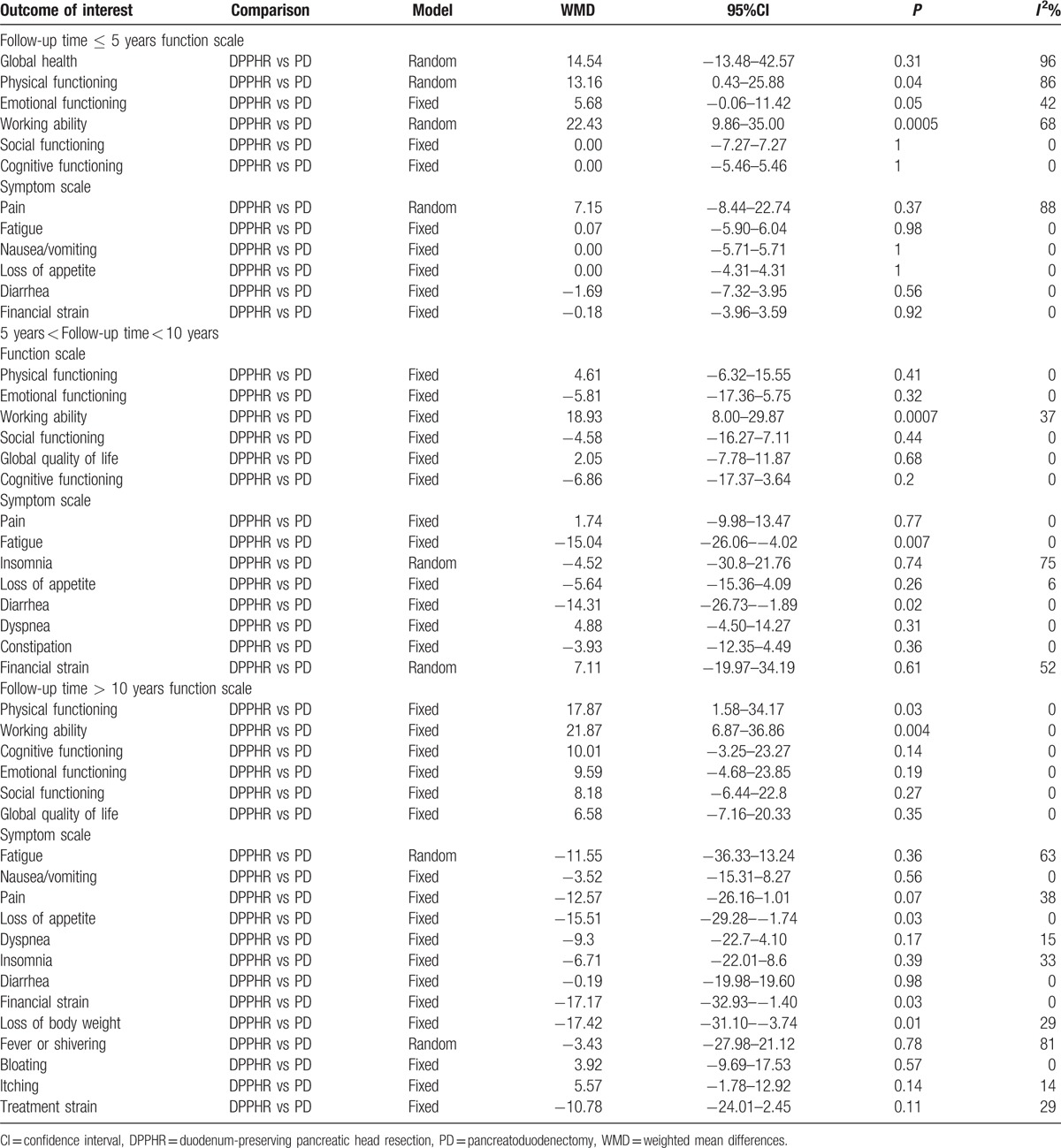
Results of different follow-up period functioning scale scores and symptom scale scores of surviving patients.

##### 3.4.7.2 The Beger vs Frey procedures

Two studies compared the Beger procedure with the Frey procedure on quality of life.^[23,24]^ Both the Beger and Frey procedures ensured a comparable quality of life (WMD = −9.00, 95% CI = −21.70 to 3.70, *P* = 0.16).

#### 3.4.8 Long-term outcomes: occupational rehabilitation

##### 3.4.8.1 DPPHR vs PD

**Figure 15:**
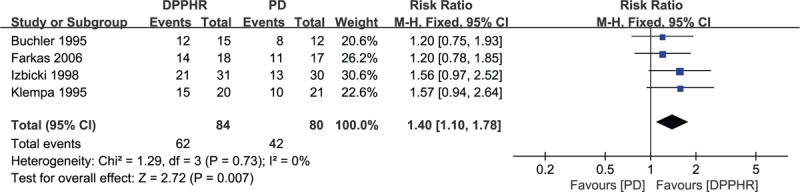
Forest plot of randomized controlled trials of duodenum-preserving pancreatic head resection versus pancreaticoduodenectomy in occupational rehabilitation.

Four studies reported data on professional or occupational rehabilitation and included 164 patients.^[18,19,21,22]^ Patients received follow-up to evaluate whether they were fully rehabilitated and able to return to work. Patients who underwent DPPHR were exposed to a significantly better rehabilitation (RR = 1.40, 95% CI = 1.10–1.78, *P* = 0.007) (Fig. 15).

##### 3.4.8.2 The Beger procedure vs PD

**Figure 16:**
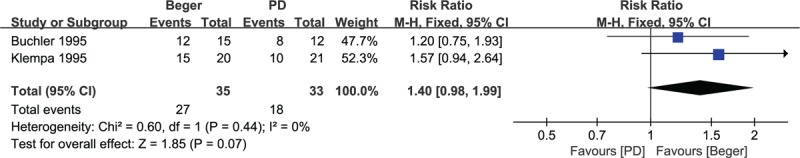
Forest plot of randomized controlled trials of the Beger procedure versus pancreaticoduodenectomy in occupational rehabilitation.

Two studies compared the Beger procedure with PD^[19,21]^ and reported data on occupational rehabilitation, which showed that there were no significant differences between the 2 groups in occupational rehabilitation (RR = 1.40, 95% CI = 0.98–1.99, *P* = 0.07) (Fig. 16).

#### 3.4.9 Subgroup and sensitivity analysis

Subgroup analyses were performed to evaluate whether the RRs of outcome measures were different among the various surgical techniques as well as the duration of follow-up. Due to the quality of the studies included in the meta-analysis, which were low to moderate, a sensitivity analysis had to be performed to test the robustness of the methodologies and to assess the stability of the pooled results. Among the 7 included RCT studies, only 1 was German and exclusion of this study had no effect on the outcomes. Among the majority of the studies, the observed results of significance were not obviously changed after the sequential omission of each study.

## 4 Discussion

### 4.1 Summary of the main results

Intractable pain and patients who had failed conservative or endoscopic therapies were the most common surgical indications for chronic pancreatitis (CP). With the rising incidence of CP, definitive management is greatly needed. Currently, PPPD and DPPHR procedures have been the standard of care for the treatment of CP.^[30,31]^ Therefore, the focus of this analysis was to evaluate the efficacy of 2 differing surgical approaches in pancreatic surgery for the treatment of chronic pancreatitis.

The updated meta-analysis identified 7 published studies that assessed the outcomes of patients with CP who underwent DPPHR or PD, and compared the surgical procedure (DPPHR vs PD or Beger vs Frey). For the main results, all procedures are equally effective for the management of pain and endocrine insufficiency for chronic pancreatitis. Improved short-term outcomes including operative times, blood transfusions, hospital stays, and postoperation morbidities were detected in patients who underwent DPPHR. For the outcome of operative time, blood transfusion, hospital stay, the studies by Farkas et al^[18]^, Izbicki et al,^[22]^ and Keck et al^[20]^, showed that the DPPHR procedure had a distinct advantage in these aspects.^[18]^ The reason might be because the DPPHR procedure had preserved the duodenum and other surrounding organs, was relatively easy, and resulted in less trauma to the patient.

As for the outcome of postoperative morbidity, the results in the previous study by McClaine et al^[32]^ showed that there were no differences in 30-day morbidity between DPPHR and PD. However, the pooled analysis indicated a significantly low incidence of postoperative morbidity in the DPPHR group, which was consistent with previous studies.^[33]^

When evaluating the outcome of endocrine and exocrine insufficiency, the study conducted by Malfertheiner et al^[34]^ indicated that the duodenum played an important role in the regulation of pancreatic polypeptide secretion and release after meals. The DPPHR procedure is performed by preserving the duodenum and other surrounding organs, with less portions of pancreatic tissue that are removed to better retain both the endocrine and exocrine functions of the pancreas. The difference in endocrine insufficiency was not significant between groups. In theory, the endocrine function should not be affected because the islets of Langerhans that contain β-cells are distributed predominantly in the tail of the pancreas.^[35]^ Patients who underwent a DPPHR procedure reported a better outcome of exocrine insufficiency (follow-up time < 60 months) when compared with patients who underwent a PPPD procedure. The Malka D's study in 2000 suggested that long-term development of pancreatic insufficiency was not reliant on the type of surgical procedure, but instead may be related to the features of CP.^[36]^ This may be due to the slightly larger remaining pancreatic tissue left over after DPPHR, which could retain better exocrine function, whereas, this advantage of DPPHR does not exist in a follow-up period of more than 60 months. It indicated that with an extension of follow-up time, the endocrine and exocrine functions of the retained part of pancreas will gradually decrease.

The main goal of operative therapy for patients who suffer from chronic pancreatitis is the relief of abdominal pain. The results of this meta-analysis showed there were no obvious differences between the PD and DPPHR procedures in pain relief. When the Beger and Frey procedures were compared within a mean follow-up of 1.5 years, pain relief was described in 95% of Beger's group and in 89% in Frey's group. No significant differences were found between the 2 groups. These findings support the implication of inflammatory pancreatic head involvement in causing intractable pain because both procedures include pancreatic head resection. If the pancreatic lesion can be removed, then the patient's pain can be alleviated.

Improved intermediate and long-term outcomes including quality of life, weight gain, and occupational rehabilitation make DPPHR a more favorable surgical strategy in patients with chronic pancreatitis. In DPPHR, the pylorus, duodenum and extrahepatic bile duct are preserved; thus, DPPHR has an obvious advantage over PD in the long-term outcomes after follow-up for more than 10 years. Several RCTs comparing DPPHR and PD for the treatment of CP have confirmed this result.^[28,29]^

### 4.2 Comparison with previous studies

Given the long history of PD and its widespread application, the safety of these surgeries has obviously improved. Sohn et al^[37]^ reported that the operative mortality was less than 3%, whereas another single-center and large sample study conducted by Beger et al reported that the mortality rate of DPPHR was 0.8%. Both of these studies confirmed the safety of the DPPHR operation.^[38]^

In 2016, a recently completed systematic review of the surgical treatment of CP that included 5 trials revealed that DPPHR may result in a shorter hospital stay than PD, but that there was currently no evidence of any difference in mortality, adverse events, or quality of life between DPPHR and PD.^[16]^ Another meta-analysis conducted by Sukharamwala et al^[39]^ demonstrated that DPPHR was a more favorable approach than PD for patients with chronic pancreatitis.

In early 1999, Schwarz and colleagues^[40]^ concluded that DPPHR should be recommended as an effective surgical method, but that the PD procedure should be considered if malignancy was suspected or if a patient suffered from persistent pain after DPPHR. With the surgical treatment for chronic pancreatitis, more attention has been paid to the selection of operative systems in recent years. A general consensus exists that DPPHR is a favorable choice for the treatment of CP,^[41–43]^ and although there are several RCTs that compare PD with DPPHR, the results of this study highlight several points regarding the management of pancreatitis.

To our knowledge, this is an updated systematic review and meta-analysis to compare different surgical procedures for the treatment of CP. Through the literature search, there have been approximately 7 meta-analyses in recent years that compare different surgical methods (PD/PPPD, DPPHR, the Beger procedure, and the Frey procedure) for the treatment of chronic pancreatitis,^[16,40–42,44]^ although similar conclusions were obtained. To provide more evidence for clinical decision-making, this study incorporated the updated randomized controlled trials with different follow-up times and a more detailed analysis (i.e., long-term quality of life) that was assessed in the functional and symptom scale scores of surviving patients. Both scales included global health, physical functioning, emotional functioning, working ability, social functioning, cognitive functioning, pain, fatigue, nausea, loss of appetite, diarrhea, financial strain, fever, itching, treatment strain, and so on, which were analyzed using the available data which the previous study lacks. Moreover, some new comprehensive results were also observed. DPPHR offered additional advantages over PD procedures and significantly reduced the incidence of diarrhea and fatigue. Overall, analyzing the patients with follow-up periods of more than 10 years revealed obviously better results after a DPPHR procedure in terms of working ability, physical status, loss of appetite, loss of body weight, and financial strain. Therefore, the results of different follow-up times in functional and symptom scale scores lend new evidence that DPPHR is a more favorable surgical strategy for patients with chronic pancreatitis.

### 4.3 Limitations of the study

However, despite a comprehensive analysis, certain limitations of our meta-analysis should be described. First, the number of RCTs included in the meta-analysis was small. Second, in the literature-included studies, the follow-up time gap was large and was from 12 to 182 months, although a subgroup analysis was adopted to avoid bias. Third, the results for pain relief from each study was assessed by the pain score, which was self-described by patients and may provide inevitable bias. Fourth, for the portions of the studies that did not directly provide means and standard deviations, the author used Hozo's algorithm to estimate those values; this may have introduced bias. Moreover, clinical and methodological heterogeneities were seen in several parameters in the meta-analysis, given the variation in surgical techniques, patient composition, and preferences among different centers. Finally, the assessment indexes indices of the postoperative clinical complications were not unified, and differences existed in with each operative technique, such as operation surgical skills, incision length, and operation time might also affect the results. In the future, more large, high-quality clinical trials that compare the surgical approach should be expected and we will conduct a more detailed subgroup analysis to explore the sources of heterogeneity to obtain a more reliable conclusion.

## 5 Conclusion

In summary, based on short- and long-term outcomes, this study demonstrated that improved short-term outcomes, including operation time, blood transfusion, hospital stay, and postoperative morbidity, were detected in patients who underwent DPPHR. With more comprehensive results, DPPHR has an obvious advantage over PD in terms of a better quality of life. In addition, a more detailed analysis of the parameters in functional and symptom scale scores provided satisfactory results in working ability, emotional function, fatigue, diarrhea, physical status, appetite, weight gain, and financial strain for the patients in the DPPHR group. Thus, the DPPHR procedure is a more favorable surgical strategy for patients with chronic pancreatitis. Further, relevant trails are needed to prove these findings.
